# Longitudinal associations among team orientation, leadership, and teamwork behaviors in community-based school management

**DOI:** 10.1371/journal.pone.0357007

**Published:** 2026-08-27

**Authors:** Takuya Yoshida, Hiroyuki Yoshizawa, Ryosuke Asano

**Affiliations:** 1 Faculty of Education, Gifu Shotoku Gakuen University, Japan; 2 Graduate School of Education, Gifu University, Japan; 3 Faculty of Literature, Kurume University, Japan; Ho Chi Minh City University of Medicine and Pharmacy, VIET NAM

## Abstract

As securing parents’ participation in elementary school activities is not guaranteed, identifying the factors that encourage such cooperation is necessary. We examined how Japanese parents’ perceptions of parent-school-community cooperation (team orientation) and leadership related to their teamwork behaviors. We conducted a three-wave longitudinal survey of parents with children in the first, third, and fifth grades (*N* = 1,753; 51.6% female; mean age = 41.5 years, *SD* = 6.1). A random intercept cross-lagged panel model was used to examine the within-person and between-person associations among the three teamwork constructs. At the within-person level, results revealed cross-lagged effects: deviations from individuals’ expected scores in team orientation and leadership were associated with deviations from their expected teamwork behaviors at the next time point. However, deviations in teamwork behaviors were not associated with subsequent deviations in team orientation. At the between-person level, mutual associations were confirmed among all three variables. These findings suggest that parents’ perceptions of cooperative relationships among parents, teachers, and local residents, together with supportive leadership, encourage subsequent cooperative behavior in school activities.

## Introduction

Educational activities in elementary schools are supported not only by teachers but also by parents and local community members. In Japanese elementary schools, parents and local residents participate as volunteers in school activities, such as watching children go to and from school and helping them learn during class [1]. Although parental support is essential for schools [2], the population of parents turns over every year as children enroll and graduate. Furthermore, some parents are reluctant to participate in school activities. Consequently, the availability of parents willing to take on such roles is unstable. Therefore, identifying the factors that promote parental participation is necessary for developing strategies to sustain parental involvement in school activities.

This study focuses on the parents’ perceptions of teamwork among parents, teachers, and local residents. Based on the theory of teamwork [3,4], team orientation (also referred to as “a sense of cohesiveness as a team”) and supportive leadership should encourage cooperative behaviors. In Japanese elementary schools, parents’ perceptions of team orientation among these three stakeholders, as well as perceived supportive leadership from parent organization leaders, may encourage them to engage in cooperative behaviors in school activities. Thus, we investigated the reciprocal associations among parents’ perceived parent-school-community relationships, perceived supportive leadership, and cooperative behaviors.

### School-community collaboration in Japan

School education relies on collaboration among teachers, parents, and local residents. In the United States, school-community partnerships are grounded in the theory of overlapping spheres of influence [5]. According to this theory, children learn and grow in three primary contexts: home, school, and community. Thus, collaboration among the three may promote children’s learning and development.

Although the form of community collaboration in Japanese schools differs from that in the U.S., both systems rely on cooperation among parents, teachers, and local residents to promote children’s learning and development. While community collaboration in the West typically refers to support from local institutions, such as libraries and hospitals [6,7], the Japanese model focuses on involving parents and local residents in school management and volunteer activities [8]. Parents and local residents undertake a variety of roles, such as watching children go to and from school, reading to them and supporting their learning in class, visiting classes, and organizing athletic events. In Japan, most parents of elementary and junior high school students join the parent-teacher association (PTA), which organizes parental support for schools. The PTA president serves as the organization’s representative, liaising with external organizations such as local governments and boards of education and exchanging views on the PTA’s behalf. Therefore, cooperation among families, schools, and communities is expected to enhance Japanese children’s learning and development, as in the U.S. [9].

Thus, clarifying the factors that promote parental cooperation is necessary to sustain parental involvement in school activities. School environment is one such factor. School environmental factors represent the degree to which schools seek cooperation from parents [10]. Previous studies have examined factors that encourage parental involvement in school activities, such as school’s recognition of the value of parental involvement in children’s learning, invitations to participate in school events, and requests for help with children’s homework [11,12].

Although the school environment promotes parental involvement, previous studies have not addressed parent-school-community partnerships. In Japanese elementary schools, local residents and parents participate in school activities; thus, the relationships among parents, teachers, and local residents may influence parental involvement in schools. Therefore, addressing the parent-school-community partnership as a school environmental factor and clarifying its impact on parental participation in school activities is important. This study examined the impact of school-community cooperation from the perspective of teamwork theory. Given the Japanese form of parent-school-community partnerships, parents’ perceptions of the relationship among parents, teachers, and local residents may act as a determinant of school participation.

### Teamwork for community-based school management

This study focused on teamwork as a theoretical framework to capture cooperation among parents, teachers, and local residents. Teamwork comprises three components: behavioral, cognitive, and leadership. It represents the shared cognitions and behaviors universally necessary for any team to accomplish specific tasks that correspond to team goals [3,4,13]. When team members have common goals, they communicate closely, flexibly adjust their roles, and support each other, which implies good teamwork and leads to high performance [3,4].

Yoshida et al. [14] developed the Teamwork Inventory for Community-based School Management (TICSM) to capture the cooperation among parents, teachers, and local residents in Japanese elementary schools. This scale is based on Dickinson and McIntyre’s theoretical framework [3] and comprises three factors: teamwork behaviors, team orientation, and team leadership. Teamwork behaviors are referred to as “team processes.” However, to avoid confusion with the process of teamwork formation examined in this study, we consistently use the term teamwork behaviors. These behaviors include five components: monitoring, feedback, supportive behavior, mutual adjustment, and communication [3]. Monitoring refers to the behavior of tracking each other’s work progress and understanding the team’s current status. Feedback refers to the behavior of providing each other with information and suggestions to solve work problems. Supportive behavior refers to the behavior of providing assistance among members, while mutual adjustment refers to the behavior of coordinating overall activities according to work progress. Communication represents behaviors related to the transfer of information among team members. Teams that are more proactive in these behaviors exhibit superior performance [15]. In the context of school-community cooperation, teamwork behaviors involve awareness of both individual actions and actions taken by the school as a whole.

Team orientation represents the attitude of maintaining good interpersonal relationships and actively working on tasks and shared goals [3]. It includes shared norms within the team, a high degree of cohesiveness, and recognition of the importance of being a part of a team [3]. Team orientation is an important foundation of teamwork and is associated with the behavioral components that constitute a team process [16,17]. Team orientation is often treated as an individual trait characterized by a cooperative disposition or a preference for group goals [17]. However, team orientation, as measured by the TICSM, is an evaluation of the team to which one belongs, rather than an individual trait. Hence, team orientation reflects the perception that parents, teachers, and local residents are willing to cooperate and actively engage in school activities for their children.

Leadership is the third component of teamwork. Team leadership refers to the influence that a leader exerts on team members’ interactions, such as providing direction and support [3]. Although these behaviors overlap with teamwork behaviors, team leadership differs in that it captures an evaluation of the leader’s own behavior, rather than the behavior of team members in general. Team leadership, similar to team orientation and teamwork behaviors, is another variable that affects team performance [18]. Leadership facilitates teamwork behaviors and improves team performance [19]. Who is identified as the leader depends on the respondent’s role. For teachers and local residents, the equivalent is a principal or vice principal and head of the community organization, respectively. Furthermore, for the parents targeted in this study, the leader was the president or vice president of the PTA. In Japanese elementary schools, the PTA president and vice president are typically parents.

Both parent and teacher versions of the TICSM have been developed and validated [14,20]. Furthermore, parent-school-community teamwork is associated with children’s learning. Yoshida et al. [21] reported that parent-school-community teamwork at elementary and junior high schools was positively associated with children’s motivation to learn and classroom adjustment, as rated by teachers. This suggests that enhancing parent-school-community cooperation in elementary schools benefits children.

To date, the longitudinal and temporal relationships among the elements of teamwork in school management have not yet been fully clarified. Identifying the temporal sequencing among the elements of teamwork is theoretically important and practically useful, as it can inform interventions to enhance teamwork in school settings. This study uses longitudinal data to examine the reciprocal relations among the components of teamwork in order to gain insights into causal relationships.

### Reciprocal relations among the components of teamwork

Among the three components of teamwork, team orientation may be positively associated with teamwork behaviors. This is because when parents sense cooperative relationships among themselves, between parents and teachers, and between parents and community members, they are likely to want to play an active role within that team and will actually engage in cooperative behavior. Kilcullen et al.’s [17] meta-analysis reported that team orientation was positively associated with teamwork behaviors at both individual and team levels. The individual level represents individuals’ tendency to emphasize the achievement of group goals as a characteristic trait, regardless of the team’s goals. The team level indicates the team’s tendency to emphasize the achievement of group goals. Since team orientation has a motivational component, a stronger desire to cooperate for the team should correspond to greater cooperative behavior. Even parents who do not actively cooperate in school activities can observe or learn about what is happening at school through newsletters. If parents find participating in school activities alongside teachers and community members enjoyable, they will be more motivated to engage in school activities. The more individuals perceive a school to be team-oriented, that is, the more cohesive the school is, the easier it will be to recognize that the school as a whole is effectively engaging in teamwork. Therefore, team orientation may be a predictor of teamwork behaviors.

Team leadership, similar to team orientation, may also be positively related to teamwork behaviors. This is because cooperative behaviors toward the team is likely to be encouraged under a leader who manages interpersonal and material resources and promotes flexible responses. Zaccaro et al. [19] provided a conceptual framework for the process by which team leadership influences team performance through teamwork behaviors. According to this framework, leaders must fulfill four functions to achieve effective results: searching for and structuring information, using information in problem solving, managing personnel resources, and managing material resources. Since team leadership in the TICSM reflects these functions, we expected team leadership to influence teamwork behaviors.

In this study, we used the RI-CLPM [22], an extension of the traditional cross-lagged panel model (CLPM), to estimate the longitudinal associations of team orientation and leadership with teamwork behaviors. This methodology distinguishes between-person differences from within-person variance within the cross-lagged panel framework [22]. Although the RI-CLPM does not fully prove causality, it strengthens temporal inference. The CLPM is a common statistical analysis method used to verify the directionality of effects. However, it has been criticized for being unable to distinguish between within-person and between-person effects [22]. In contrast, the RI-CLPM is well suited for analyzing longitudinal data as it separates between-person and within-person stability over time. The RI-CLPM partitions the time-invariant part as the random intercept, which represents people’s trait-like individual differences. The autoregressive paths reflect the extent of within-person variances. Cross-lagged paths reflect the extent to which a within-person deviation on one variable at one time point is related to a within-person deviation on a different variable at the next time point, controlling for autoregressive effects and the concurrent association between variables. Therefore, we employed the RI-CLPM to examine the within-person-level processes across the three factors of teamwork among parents, teachers, and local residents.

### Present study

This longitudinal study examined the reciprocal associations among three factors related to teamwork among parents, teachers, and local residents. Due to the importance of parents’ sustained participation in school activities, we targeted the parents of Japanese elementary school students. Specifically, we conducted three-wave surveys of parents with eldest children in the first, third, and fifth grades over one year to examine reciprocal relations among the components of teamwork. Since previous studies on “parental involvement”—a concept closely related to school-community cooperation—have focused on changes occurring within a few months [23,24], we set the survey interval at four months.

Regarding the relationships among the components of teamwork at the within-person level, we predicted that team orientation and team leadership would positively relate to teamwork behaviors. Furthermore, we predicted a between-person-level association, such that individuals who perceive higher levels of team orientation and team leadership would be more likely to engage in positive teamwork behaviors. Because time-specific effects were considered unlikely, we used equality constraints to test whether the paths were consistent across measurement waves.

Thus, we formulated the following hypotheses:

*Hypothesis 1:* Team orientation predicts subsequent teamwork behaviors at the within-person level.

*Hypothesis 2:* Team leadership predicts subsequent teamwork behaviors at the within-person level.

In this study, we treat both team orientation and team leadership as antecedents of teamwork behaviors and do not assume a specific order between them. Previous research has shown that cooperative and supportive leadership styles are positively correlated with team orientation [25–27]. Based on this, while no association is expected between team orientation and team leadership at the within-person level, a positive association is likely to be observed at the between-person level.

## Methods

### Participants and procedures

A web-based survey was conducted among the parents of first-, third-, and fifth-grade children who attended public elementary schools. Ideally, the survey would have included parents of children in all grades from first through sixth grade; however, due to budget constraints, we limited the survey to first, third, and fifth graders to cover the lower, middle, and upper elementary grades. Participants were recruited through Cross Marketing, Inc., ensuring a sample size of at least 3,000 at Time 1. Based on the research firm’s projection of the dropout rate, the target sample size for Time 3 was set at 900. Surveys were conducted in June 2021 (Time 1), October 2021 (Time 2), and February 2022 (Time 3). Assignments were made so that the eldest child’s grade and parents’ gender were evenly distributed. The research firm restricted the target age range to 20–59 years. To ensure that respondents read the questions carefully, we included two directed (attention check) questions [28] among the survey items (e.g., “Please answer this question with ‘I do not agree at all’”) at each time point; only those who passed these questions were eligible. Data were collected from 3,039, 1,930, and 988 parents at Times 1, 2, and 3, respectively. In addition, the survey included items asking about the leadership of PTA presidents and vice presidents, and respondents were instructed to answer “neither agree nor disagree” for all items if they had never met the PTA president or vice president. Therefore, we excluded from the analysis those who gave an identical “neither agree nor disagree” response to every leadership item at Time 1. Consequently, 1,286 of the 3,039 respondents surveyed at Time 1 were excluded from the analysis. The number of respondents included in the analysis was 1,753 for Time 1, 1,115 for Time 2, and 588 for Time 3. Of the respondents at Time 1, 905 were mothers and 848 were fathers. Their mean age was 41.5 years (*SD* = 6.1).

Table 1 presents the number of participants who provided data and were analyzed. Chi-square (χ^2^) tests were conducted to compare participants at Times 1, 2, and 3. No significant differences were observed in the distribution of gender [χ^2^(2) = 1.83, *p* = .40], children’s grade [χ^2^(4) = 4.71, *p* = .32], or perceived socio-economic status [χ^2^(8) = 7.18, *p* = .52]. We examined whether there were differences in the scores on the three measures of teamwork between participants who dropped out and those who did not. Specifically, we conducted a one-way ANOVA comparing three groups: those who responded only at Time 1, those who responded through Time 2, and those who responded at all three time points. No significant *F* value was observed for teamwork behaviors [*F* (2, 1750) = 1.19, *p* = .31]. No significant *F* value was observed for leadership either [*F* (2, 1750) = 2.82, *p* = .06]. A significant F value was observed for team orientation [*F* (2, 1750) = 4.00, *p* = .018]. A post hoc multiple comparison using the Holm method revealed that the team orientation scores of participants who completed only Time 1 (*M* = 3.16, *SE* = .03) were lower than those of participants who completed both Time 1 and Time 2 (*M* = 3.28, *SE* = .03) (*p* = .014). However, the effect size was very small (partial η² = .005). Therefore, although some caution is warranted regarding the team orientation scores, there was no clear evidence of systematic attrition bias.

**Table 1:** Demographic characteristics of participants.

		Time 1		Time 2		Time 3	
Gender of the parent (%)	Female	905	51.6%	596	53.5%	295	50.2%
	Male	848	48.4%	519	46.5%	293	49.8%
Eldest child’s grade (%)	First	495	28.2%	291	26.1%	143	24.3%
	Third	614	35.0%	405	36.3%	208	35.4%
	Fifth	644	36.7%	419	37.6%	237	40.3%
Mean age (*SD*)		41.50	(6.10)	41.79	(5.95)	42.43	(5.88)
Mean years of residence (*SD*)		11.63	(10.63)	11.55	(10.61)	12.68	(11.47)
SES (*SD*)		3.01	(0.85)	3.03	(0.82)	3.07	(0.82)

Note: SES: socio-economic status. SES was self-reported on a scale from 1 (low) to 5 (high).

Statistical power was calculated using the R package powRICLPM [29]. We conducted 1,000 Monte Carlo simulations. Referring to Yoshida et al. [14], who assessed the association among the three TICSM factors, we assumed that the within-person correlation between team orientation, team leadership, and teamwork behaviors at the same time points was *r* = .60. For unknown associations and effects, we referred to Orth et al.’s effect size criterion [30]. We used the following specifications: β = .12 (large) for both the autoregressive effect and the cross-lagged effect; proportion of between-unit variance = .50; and *r* = .35 (moderate) for the correlation between the random intercepts. Results of the Monte Carlo simulation revealed that our sample size (*N* = 1,700) demonstrated a power of 49.6%. Because this is well below the conventional 80% threshold, statistical power may not have been sufficient.

### Measures

During the survey’s screening, respondents were asked whether their eldest child attended a public elementary school and, if so, the child’s grade. Demographic characteristics, such as age, gender, occupation, income, and location, were obtained from the survey panel’s registered respondent profile data.

Subjective socio-economic status (SES) was measured via a single item [31]. Participants were asked the question “If Japanese society as a whole were divided into five strata, into which stratum would you place yourself?” Responses included 1 (low), 2 (lower-middle), 3 (middle), 4 (upper-middle), and 5 (high). Higher scores indicated a higher level of subjective SES.

The TICSM was used to assess teamwork [14]. This scale comprises 31 items on three factors: team orientation (13 items), team leadership (8 items), and teamwork behaviors (10 items). Regarding team orientation and teamwork behaviors, participants were asked, “What are your feelings about the school your eldest child attends?” Regarding team leadership, participants were asked, “What are your thoughts on the PTA president and vice president at the school your eldest child attends?” Items were rated on a 7-point Likert scale ranging from 1 (strongly disagree) to 7 (strongly agree).

The TICSM was developed in Japanese. An English-language appendix summarizing the original scale development paper [14] is available online at https://doi.org/10.17605/OSF.IO/NXQ3E. This appendix includes the theoretical background, the item development process, the results of factor analyses, and a complete list of English-translated items with factor loadings.

### Ethical considerations

This study was approved by the research ethics committee of Gifu Shotoku Gakuen University (Study Code: 2020−15). The study’s aim and an informed consent form were presented on the cover page of the online questionnaire. Participants were informed that their participation was voluntary, and they had the option to withdraw at any time. Data were completely anonymous.

### Data analysis

A longitudinal confirmatory factor analysis (CFA) was performed to confirm measurement invariance over time by testing whether the three-factor structure of the TICSM fit the data well at all three measurement points. Following Putnick and Bornstein [32], we verified measurement invariance in four steps. First, we assessed a configural invariance model based on the three-factor model. This served as the baseline model, with no equality constraints imposed. Subsequently, we examined a weak (metric) invariance model, by constraining item factor loadings to be equal over time. Third, we assessed a strong (scalar) invariance model, in which both item factor loadings and item intercepts were held equal over time. Finally, in addition to the strong invariance model, we tested a strict model in which residual variances were additionally constrained to be equal across time. Model fit was assessed using the comparative fit index (CFI; > .90), root mean square error of approximation (RMSEA; < .08), and standardized root mean square residual (SRMR; < .08); χ^2^ values, which are sensitive to sample size, are reported for reference only [33]. Cut-off values of a −0.01 change in CFI and a 0.015 change in RMSEA, respectively, were used as criteria for model comparison [34].

In the RI-CLPM, missing data were handled using full information maximum likelihood (FIML) estimation with robust standard errors. This approach yielded reliable estimates and enabled the use of all available data. To assess the RI-CLPM, we followed Hamaker et al.’s procedure [22] and compared the unconstrained model (Model 1) with a model in which the autoregressive paths were constrained to be equal over time (Model 2), and a further model in which both the autoregressive and cross-lagged paths were constrained to be equal over time (Model 3). Model fit and comparisons were performed using the recommended fit indices.

Longitudinal CFA and RI-CLPM analyses were conducted using Mplus 8.7 [35], and preliminary data analyses were performed using R 4.5.1 [36]. Raw datasets and the analysis syntaxes are available online at https://doi.org/10.17605/OSF.IO/NXQ3E.

## Results

### Descriptive statistics and correlations

Table 2 presents the descriptive statistics, reliability of the three teamwork factors at each time point, and correlations. Correlations of each teamwork factor across three waves were significant (ranging from  .45 to  .78, *ps* < .001).

**Table 2:** Means, standard deviations, alpha reliability, and zero-order correlations.

	1.	2.	3.	4.	5.	6.	7.	8.	9.
1. Team Orientation_T1									
2. Team Leadership_T1	.66								
3. Teamwork Behaviors_T1	.69	.64							
4. Team Orientation_T2	.75	.57	.58						
5. Team Leadership_T2	.54	.68	.50	.67					
6. Teamwork Behaviors_T2	.63	.58	.64	.78	.64				
7. Team Orientation_T3	.70	.50	.54	.75	.56	.62			
8. Team Leadership_T3	.54	.58	.45	.59	.68	.55	.70		
9. Teamwork Behaviors_T3	.58	.52	.58	.65	.59	.69	.75	.68	
Mean	3.21	3.12	2.88	3.21	3.13	2.88	3.20	3.10	2.86
*SD*	0.75	0.90	0.72	0.76	0.83	0.73	0.78	0.85	0.75
*α*	.94	.96	.90	.94	.96	.90	.95	.97	.91

Note: T1 = time 1, T2 = time 2, T3 = time 3. All correlations are significant at *p* < .01.

### Longitudinal CFA

A longitudinal CFA was conducted to verify measurement invariance over time. We compared four different models: the configural invariance, weak invariance, strong invariance, and strict models [32]. Table 3 summarizes the results. The configural invariance model provided an acceptable fit to the data. Subsequent models also demonstrated acceptable changes in model fit: the weak invariance model (ΔCFI = .000, ΔRMSEA = .000), the strong invariance model (ΔCFI = .000, ΔRMSEA = .000), and finally, the strict model (ΔCFI = −.003, ΔRMSEA = .000). Therefore, a strict model was adopted. Table 4 presents the factor loadings of the strict model.

**Table 3:** Measurement invariance over time.

Model	χ^2^	*df*	CFI	TLI	RMSEA [90% *CI*]	SRMR	ΔCFI	ΔRMSEA
Configural invariance	6828.713	4056	.938	.935	0.034 [0.033, 0.035]	.036		
Weak invariance	6885.385	4118	.938	.936	0.034 [0.032, 0.035]	.041	.000	.000
Strong invariance	6962.521	4180	.938	.936	0.034 [0.032, 0.035]	.042	.000	.000
Strict	7154.968	4242	.935	.934	0.034 [0.033, 0.036]	.042	−.003	.000

Note: CFI: comparative fit index; TLI: Tucker-Lewis Index; RMSEA: Root mean square error of approximation; SRMR: standardized root mean square residual.

**Table 4:** Factor loadings for the TICSM in the longitudinal CFA.

Factors and items	Factor loadings
F1 Team orientation	
I enjoy participating in school events and meeting the children.	.640
I think it is the school’s role to bring parents and community members together.	.603
Parents and community members cooperate and participate in school activities.	.821
I feel I have a lot to learn from teachers and community members.	.778
Parents who participate in school activities get along well with each other.	.746
I feel good about participating in school activities as a parent.	.784
I feel that children learn a lot from parents and community members other than their parents through school activities.	.773
Parents can easily talk to each other.	.715
Parents and community members are actively involved in school activities.	.777
I feel children are nurtured by parents and community members of the school.	.780
Activities at this school are designed to make it easy for parents and community members to participate.	.795
I can easily talk to teachers and community members.	.729
I feel I have a lot to learn from school children.	.679
F2 Team leadership	
The PTA president or vice president gives concise and to the point instructions and comments at discussions and other occasions.	.849
The PTA president and vice president serve as effective liaisons between teachers, parents, and community members.	.887
The PTA president and vice president listen to everyone.	.879
The PTA president and vice president motivate the entire group.	.872
The PTA president and vice president treat everyone fairly.	.857
The PTA president and vice president are trusted.	.885
The PTA president and vice president work with teachers and community members.	.851
The PTA president and vice president keep me informed of what I need to know.	.851
F3 Teamwork behaviors	
Information is shared frequently among parents.	.688
We support one another so that the workload does not fall too heavily on any one parent.	.695
For events in which the school asks for cooperation, parents are informed of the significance and purpose of their participation.	.688
We discuss school activities until everyone is satisfied.	.744
Parents and community members help recruit volunteers when additional help is needed.	.685
Feedback is collected from children and parents to improve school activities.	.622
Any problems are reported immediately and shared among the parents.	.662
I actively assist parents with school activities.	.761
Parents are assigned roles with minimal dissatisfaction.	.622
Parents, teachers, and community members actively interact with one another.	.758

Note: Original items are written in Japanese.

### Random intercept cross-lagged panel model

Given sufficient measurement invariance over time, we assessed the RI-CLPM to investigate the reciprocal relationship among teamwork factors. Table 5 summarizes the results. First, the RI-CLPM was estimated without any constraints (Model 1). Model 1 had an acceptable fit to the data. In the second and third steps, time invariance was assessed by constraining autoregressive effects (Model 2) and the autoregressive and cross-lagged effects (Model 3), respectively. Both Models 2 and 3 demonstrated good model fit. CFI and RMSEA differences between Models 1 and 2 indicated that Model 2 fit the data well (ΔCFI = .001, ΔRMSEA = −.002). Likewise, CFI and RMSEA differences between Models 2 and 3 indicated that Model 3 fit the data well (ΔCFI = .000, ΔRMSEA = −.008). Thus, Model 3 was chosen, indicating that the effects of T1 on T2 were equal to the effects of T2 on T3 for both the autoregressive and cross-lagged effects.

**Table 5:** Summary of the fit indices for RI-CLPM comparisons.

Model	χ^2^	*df*	CFI	TLI	RMSEA [90% *CI*]	SRMR	ΔCFI	ΔRMSEA
Model 1 with no constraints	3.397	3	1.000	0.999	0.009 [0.000,0.042]	.008		
Model 2 with constrained lagged effects	7.886	6	1.000	0.998	0.013 [0.000,0.036]	.022	.000	−.004
Model 3 with constrained lagged and cross-lagged effects	18.560	12	.999	0.996	0.018 [0.000,0.033]	.039	.000	−.005

Note: CFI: comparative fit index; TLI: Tucker-Lewis Index; RMSEA: Root mean square error of approximation; SRMR: standardized root mean square residual.

**Fig 1:**
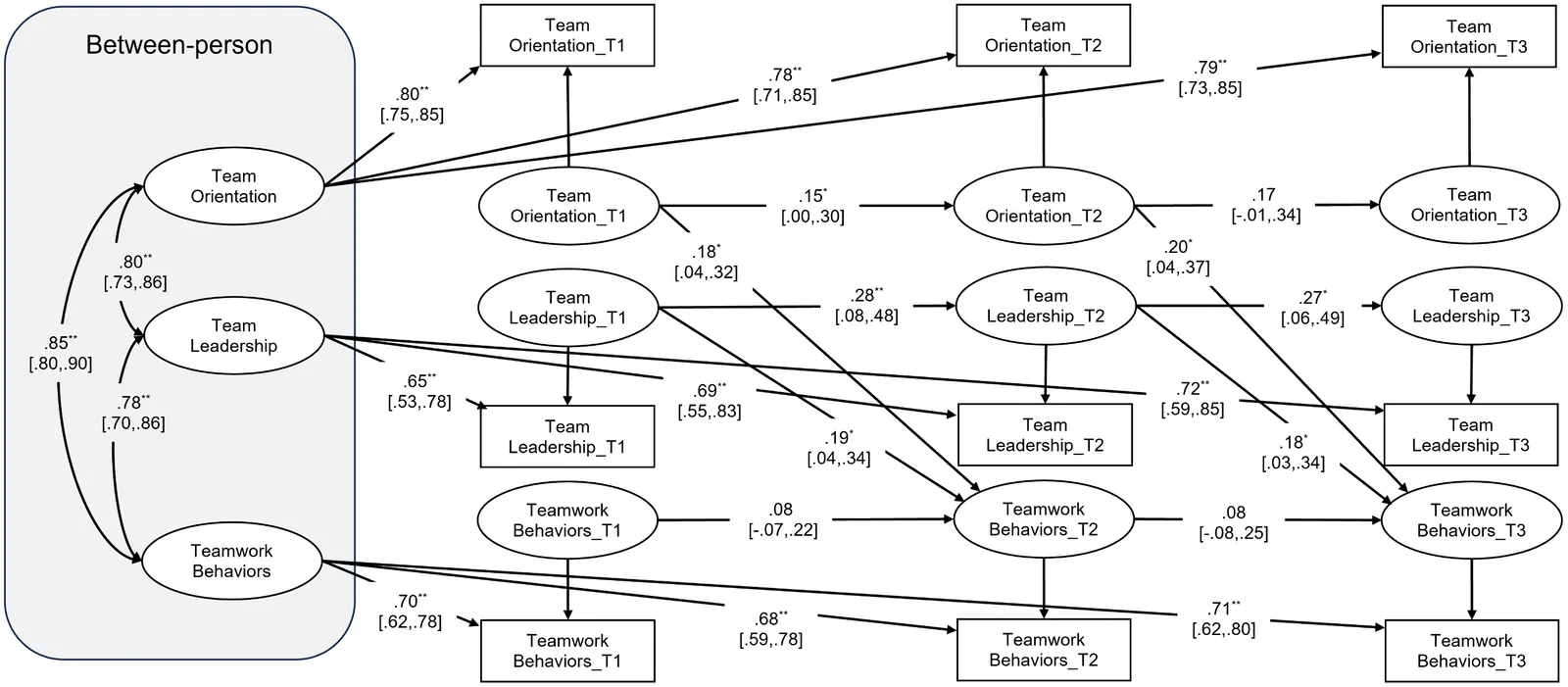
Graphic of the random-intercept cross-lagged panel model examined in the current study. T1 = time 1, T2 = time 2, T3 = time 3. Standardized coefficients are presented, with 95% confidence interval in brackets. Covariances between the variables at each wave and coefficients for non-significant cross-lagged effects were estimated but excluded from the figure for clarity. * *p* < .05, ** *p* < .01.

Fig 1 presents the results of Model 3. Regarding the within-person cross-lagged effect, team orientation significantly predicted teamwork behaviors at the next time point (b = .212, *SE* = 0.084, *p* = .012). Furthermore, team leadership significantly predicted teamwork behaviors at the next time point (b = .152, *SE* = 0.060, *p* = .011). At the between-person level, associations among the three variables were all significant and positive (bs = .23–.28, *ps* < .01).

## Discussion

This study aimed to examine the longitudinal associations among team orientation and team leadership, and teamwork behaviors via a three-wave longitudinal survey with parents of elementary school students in Japan. RI-CLPM analysis revealed two significant paths at the within-person level. Team orientation positively predicted subsequent teamwork behaviors. Furthermore, team leadership also positively predicted subsequent teamwork behaviors. Based on the effect size criteria established by Orth et al. [30], the effect sizes for the path from team orientation and team leadership to teamwork behaviors were large.

Hypothesis 1 was supported as team orientation positively predicted teamwork behaviors. Perceiving good relationships and cooperation among parents, teachers, and local residents may encourage parents to play an active role and take cooperative action. The finding that team orientation positively predicted teamwork behaviors was consistent with previous findings. Kilcullen et al.’s [17] meta-analysis reported that team orientation was positively associated with teamwork behaviors at both the individual and team levels. Although the team orientation addressed in this study does not statistically separate the individual level from the team level, it conceptually represents team-level orientation. Our results suggest that team orientation is important for promoting teamwork behaviors in school settings.

Hypothesis 2 was supported as team leadership positively predicted teamwork behaviors. This result was consistent with teamwork theory. Team leadership includes four functions, as mentioned above [19]. Leaders who perform these functions make it easier for members to understand their roles and with whom they should collaborate. Furthermore, such leaders encourage flexible responses by reassessing available resources according to the task at hand. Consequently, such leaders foster flexible and creative cooperative actions. Therefore, team leadership is likely to promote teamwork behaviors.

At the between-person level, significant positive associations were observed among all three teamwork variables. Between-person level indicates characteristic differences among individuals [22]. Individuals who generally perceive high levels of team orientation also tend to rate team leadership and teamwork behaviors more highly. Differences at the between-person level may reflect stable individual characteristics as well as variation in schools’ overall culture of cooperation: parents at schools with a strong, longstanding culture of cooperation may consistently rate team orientation, leadership, and teamwork behaviors higher. Such differences between schools may be reflected in the results at the between-person level.

### Strength and limitations

This study’s main strength is that it focuses on teamwork among parents, teachers, and local residents from the perspective of parents. To date, studies have examined several school environmental factors that promote parental involvement in school activities [11,12]. However, the relationship among parents, schools, and communities as a school environmental factor remains under researched. To the best of our knowledge, this is the first study to examine the process of teamwork formation in a school setting at both the within-person and between-person levels. We used the RI-CLPM to separate the three components of teamwork into within-person and between-person variance components. This allowed us to clarify the longitudinal associations among the teamwork components while controlling for time-invariant individual differences. Results suggest that parents’ perceptions of the relationship among parents, schools, and communities act as a determining factor in school participation.

However, this study has several limitations. First, causal conclusions cannot be drawn regarding the formation process of teamwork. Although our cross-lagged longitudinal design goes beyond cross-sectional correlations by accounting for temporal precedence, it still precludes drawing definitive causal conclusions. Furthermore, although leadership has multiple functions [19], this study treated leadership as a single construct. Therefore, it remains unclear which function of leadership is strongly associated with teamwork behaviors. Future research should include longitudinal studies comparing schools with different ways of collaboration, studies that account for the influence of school events as confounding variables, and experimental designs that compare various intervention methods to clarify causal relationships.

Second, this study lacked objective measures of performance as outcome variables. Objective indicators of the degree of school-community collaboration could include volunteer participation rate and the percentage of parents who voluntarily put themselves forward as PTA board members. Other possible effectiveness indicators of school-community collaboration may include children’s grades and the percentage of children participating as volunteers. Future empirical studies should utilize these indicators.

Third, the measured data consist solely of self-reports. We cannot rule out the possibility of social desirability bias, such as parents overestimating their own teamwork behaviors or the level of cooperation among fellow parents. Future research should incorporate peer evaluations, such as having teachers and community members assess parents’ activities.

Fourth, our sample was limited to Japanese participants. The form of school-community collaboration in Japan is unique and differs from that of other countries [6–8]. Since the nature of cooperation differs from country to country and region to region, accumulating studies that reflect the forms of cooperation in each region is necessary. In rural schools in the United States, the collaboration between schools and communities is primarily aimed at community renewal [37]. Thus, in such schools, members’ objectives and actions differ from those of the Japanese schools examined in this study. Therefore, future studies should consider these cultural differences. However, the perception of team orientation may universally promote willingness to cooperate within one’s own team or organization. Future empirical studies should clarify the connection between theory and practice.

Fifth, this study focused on fluctuations in teamwork over a relatively short period. It cannot be ruled out that teamwork behaviors may influence team orientation over the course of the six years of elementary school. A longitudinal study spanning several years would be necessary.

### Implications for theory and practice

This study contributes to teamwork theory by revealing the temporal dynamics by which teamwork forms among parents, teachers, and local residents. This study suggests a longitudinal relationship in which team orientation and team leadership promote cooperative behaviors. Few studies have examined the temporal sequencing among the elements of teamwork in the context of schools and industrial organizations. This study empirically demonstrated that perceptions of team orientation and leadership temporally precede cooperative behavior. Thus, this study provides valuable empirical evidence for the teamwork theory.

Our findings are also beneficial for practice. Traditionally, the factors that define parental involvement in school activities have focused on how parents perceive the demands placed on them by the school and by teachers [10]. The collaborative relationship among the three parties has not been emphasized. Our results suggest that it is reasonable to add parent-school-community collaboration as a school environmental factor that supports sustained parental involvement in school activities. Since team orientation and leadership were positively associated with teamwork behaviors, devising ways to enhance parents’ perceived team orientation and team leadership is important. Furthermore, increasing opportunities for parents to see how parents, local residents, and teachers work together, and how PTA presidents and vice presidents demonstrate leadership, may be beneficial.

## Conclusion

This study focused on parent-school-community collaborative relationships as perceived by parents. We used a three-wave longitudinal design and investigated how team orientation among parents, teachers, and local residents, as well as the leadership of PTA presidents and vice presidents, were related to teamwork behaviors. Results revealed that team orientation was positively associated with teamwork behaviors at the within-person level; however, teamwork behaviors did not predict team orientation. Furthermore, team leadership predicted teamwork behaviors. Our findings may contribute to sustaining participation of parental volunteers who support school activities and promote children’s learning and development. These results suggest that parents who highly value school-community cooperation at the schools their children attend are more likely to engage in school activities.
